# Compost mixed fruits and vegetable waste biochar with ACC deaminase rhizobacteria can minimize lead stress in mint plants

**DOI:** 10.1038/s41598-021-86082-9

**Published:** 2021-03-23

**Authors:** Muhammad Zafar-ul-Hye, Muhammad Tahzeeb-ul-Hassan, Abdul Wahid, Subhan Danish, Muhammad Jamil Khan, Shah Fahad, Martin Brtnicky, Ghulam Sabir Hussain, Martin Leonardo Battaglia, Rahul Datta

**Affiliations:** 1 Department of Soil Science, Faculty of Agricultural Sciences and Technology, Bahauddin Zakariya University, Multan, Punjab 60800 Pakistan; 2 Department of Environmental Sciences, Bahauddin Zakariya University, Multan, Punjab 60800 Pakistan; 3 Department of Soil and Environmental Sciences, Faculty of Agriculture, Gomal University, Dera Ismail Khan, KPK Pakistan; 4 Department of Agronomy, The University of Haripur, Haripur, Khyber Pakhtunkhwa 22620 Pakistan; 5 Department of Agrochemistry, Soil Science, Microbiology and Plant Nutrition, Faculty of AgriSciences, Mendel University in Brno, Zemedelska 1, 61300 Brno, Czech Republic; 6 Faculty of Chemistry, Institute of Chemistry and Technology of Environmental Protection, Brno University of Technology, Purkynova 118, 62100 Brno, Czech Republic; 7 Department of Technical Services, Fatima Agri Sales and Services, Bahawalpur, Punjab Pakistan; 8 Department of Animal Sciences, Cornell University, Ithaca, USA; 9 Department of Geology and Pedology, Faculty of Forestry and Wood Technology, Mendel University in Brno, Zemedelska1, 61300 Brno, Czech Republic

**Keywords:** Microbiology techniques, Plant symbiosis, Soil microbiology, Pollution remediation

## Abstract

High lead (Pb) concentration in soils is becoming a severe threat to human health. It also deteriorates plants, growth, yield and quality of food. Although the use of plant growth-promoting rhizobacteria (PGPR), biochar and compost can be effective environment-friendly amendments for decreasing Pb stress in crop plants, the impacts of their simultaneous co-application has not been well documented. Thus current study was carried, was conducted to investigate the role of rhizobacteria and compost mixed biochar (CB) under Pb stress on selected soil properties and agronomic parameters in mint (*Mentha piperita* L.) plants. To this end, six treatments were studied: *Alcaligenes faecalis*, *Bacillus amyloliquefaciens*, CB, PGPR1 + CB, PGPR2 + CB and control. Results showed that the application *A. faecalis* + CB significantly decreased soil pH and EC over control. However, OM, nitrogen, phosphorus and potassium concentration were significantly improved in the soil where *A. faecalis* + CB was applied over control. The *A. faecalis* + CB treatment significantly improved mint plant root dry weight (58%), leaves dry weight (32%), chlorophyll (37%), and N (46%), P (39%) and K (63%) leave concentration, while also decreasing the leaves Pb uptake by 13.5% when compared to the unamended control. In conclusion, *A. faecalis* + CB has a greater potential to improve overall soil quality, fertility and mint plant productivity under high Pb soil concentration compared to the sole application of CB and *A. faecalis*.

## Introduction

Heavy metals are potential toxic for humans, animals, soil microorganisms and plants^1–6^. Presence of heavy metals beyond the threshold limit in soils adversely affects crop productivity^7–9^. Among others, lead (Pb) has become one of the major soil contaminants that continuously deteriorate soil health^10,11^. Although a non-essential nutrients for plant growth, Pb in solution can relatively easily be taken up by plants in their natural environment and accumulate as an insoluble form within plants roots^12^. Once inside the plant, Pb disturbs a broad range of biochemical and physiological metabolic processes including nitrate assimilation, water status plant growth and seed germination, and this results in poor growth and development^13–16^. However, Pb translocation is limited in plants and mostly occurs from roots to shoot but not the inverse way^17^. Other physiological and biochemical attributes such as carotenoid content and activity, CO_2_ assimilation rate, and chlorophyll and photosynthetic rate, among others, are significantly decreased under over-optimum Pb concentrations in plant^13^. It is well documented that fertilizers and automobiles are major sources of Pb pollution generation^18^. Other anthropogenic activities such as i.e., burning of fossil fuels also facilitate the overaccumulation of Pb in water, air, and soil^19^. However, weathering of Pb enriched rocks, use of sewage water for irrigation purposes, shedding paint chips, use of leaded gasoline in motor vehicles and waste disposal represent additional sources of Pb accumulation in soil^20^.

Over the last 20 years, scientists around the world have investigated and further refined different strategies to reduce the Pb toxicity problem in crops^1,21,22^. Among these, biochar application has been reported as one of the most promising to ameliorate the toxic effects of high heavy metals concentrations in soils and crops^23^. Specific properties of biochar like high surface area, porosity and adsorption rates are largely dependent on the pyrolysis conditions and feedstock type, and can confer biochar with the ability to retain and sorb numerous compounds in the soil such as organic contaminants and heavy metals^23–29^. Additionally, the exchange capacity, microporous structure and active functional groups of biochar played a vital role in minimizing the bioavailability and mobilization of heavy metals^30^.

Use of organic amendments^31,32^ i.e., compost in agriculture increases rhizobacterial proliferation, water and nutrients holding capacity and soil aggregation. It also decreases soil pH when applied in soil^33^. By maintaining the soil organic pool, compost in soil enhances the phytoavailability of macro and micronutrients by improving soil health^33–35^, although foliar application of fertilizer and micronutrients as demonstrated to be a better alternative to fast action in some cases^34–38^. Several rhizobacteria have also been identified as potential biofertilizers that could have positive effects on crop quality and yield^37–45^. These rhizobacteria are found in the plant rhizosphere and are collectively known as plant growth-promoting rhizobacteria (PGPR)^44–48^. Most PGPR secretes phytohormones, mobilized nutrients in the soil, nonsymbiotically fixed nitrogen, and decreased stress ethylene in crops^46,49^. Additionally, these PGPR also enhanced systemic resistance (disease-resistance mechanisms) and proved biocontrol agents against diseases^49,50^. Plant growth-promoting bacteria (PGPB) help in the mitigation of abiotic stresses in plants^51,52^. Besides the profound positive impacts that use of biostimulants have shown in overall soil health and fertility^53,54^, use of biostimulants can also help to reduce the phytotoxicity resulting from high soil Pb concentrations^55^.

Mint (*Mentha piperita* L.), a plant that belongs to Labiatae family, is cultivated under both field and greenhouse conditions in Pakistan^56^ for the production of fresh or dried herbs and essential oils^57^. Fresh and dried mint herbs for flavoring of beverages and foods and used for teas. Mint essential oils are used on a large scale as aromatic agents in toothpaste, chewing gum, mouthwash, candy, and aromatherapy. Mint essential oils are also used in eco-friendly pesticides, antimicrobial agents and pharmaceuticals^58^. The essential oils, extracts and herbs contain a big history of medicinal usage for symptomatic and therapy treatments of numerous human disorders and diseases^59^. However, mint plants are particularly susceptible to high concentrations of heavy metals in soils, particularly Pb^60^, a heavy metal that has been reported in high concentration across several soils and ecosystems in Pakistan, according to The World Health Organization^61^. Therefore, a pot experiment was conducted to investigate the impacts of co-application of rhizobacteria in the presence and absence of mixed biochar (CB) on growth and Pb uptake in mint. It is hypothesized that co-application of rhizobacteria and CB could be an efficacious technique for alleviation of Pb toxicity in mint over sole application.

## Results

**Figure 1:**
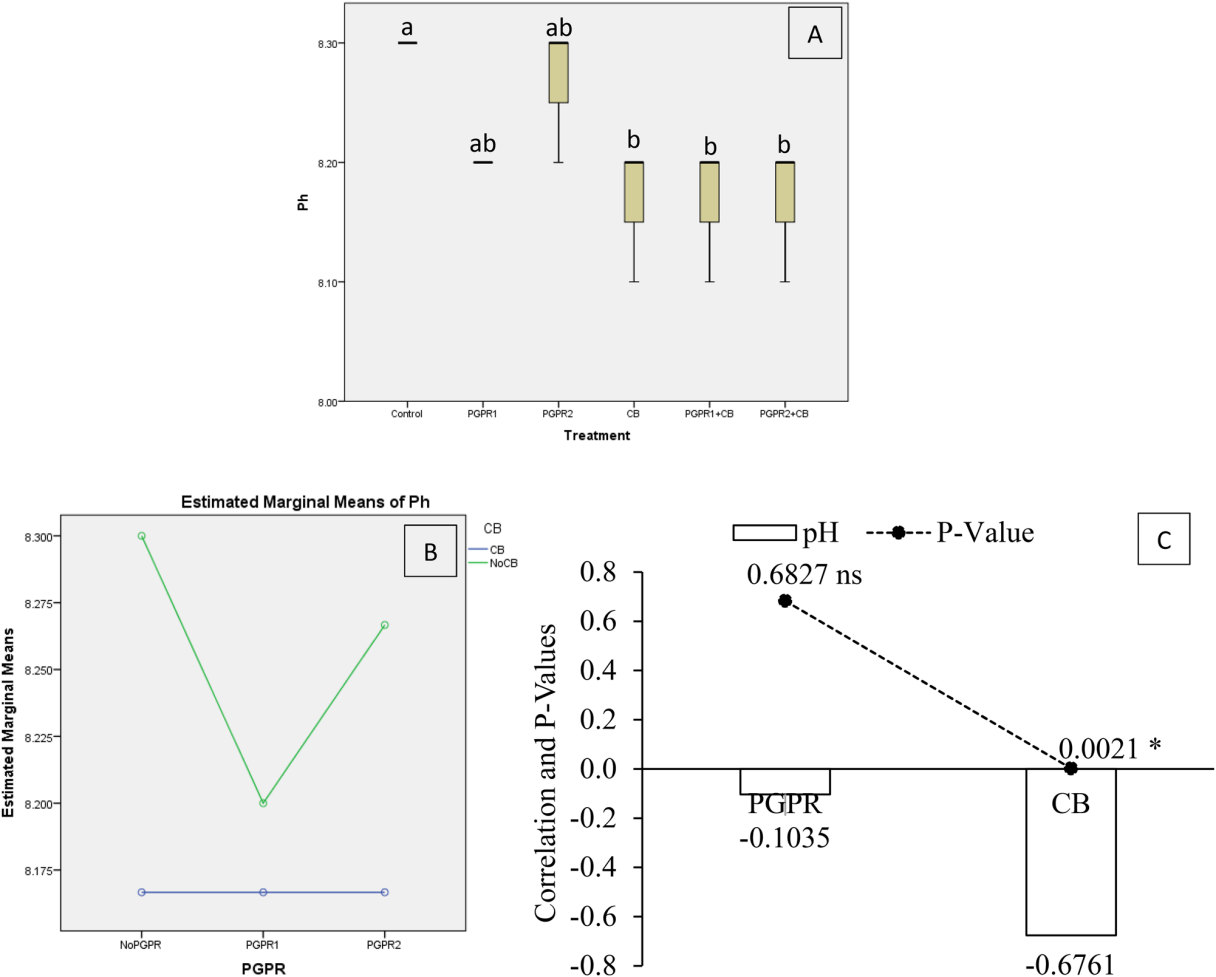
Effect of treatments on pH of soil. Values are the average of three replicates (**A**). Different letters showed significant differences (Tukey’s test; *p* ≤ 0.05). Interaction graph of PGPR and CB for soil pH*s* (**B**). Correlation graph of PGPR and CB for soil pH*s* (**C**).

The effect of treatments was significant on soil pH under artificially induced lead (Pb) stress. Interaction of PGPR and CB was non-significant but ordinal for soil pH (Fig. 1B). Results showed that PGPR1 + CB, CB, and PGPR2 + CB significantly decreased soil pH over control (Fig. 1A). No significant increase was noted over soil pH of control, where sole inoculation of PGPR1 and PGPR2 was done. It was noted that CB showed significant (0.0021) negative (− 0.6761) correlation while PGPR showed non-significant (0.6821) negative (− 0.1035) correlation with soil pH (Fig. 1C). A significant reduction of 1.63% in soil pH was observed over control in PGPR2 + CB, CB and PGPR1 + CB.

**Figure 2:**
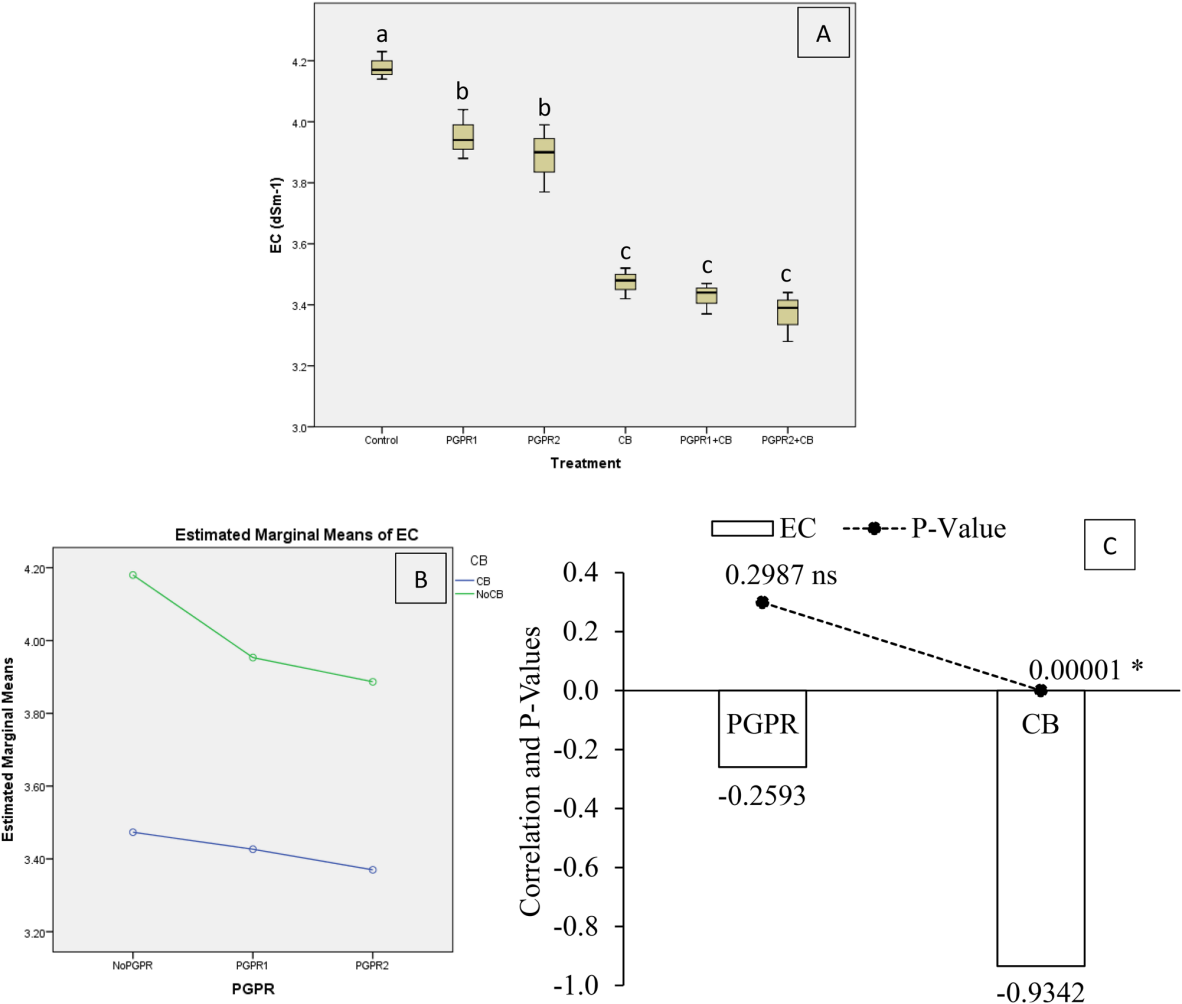
Effect of treatments on EC of soil. Values are the average of three replicates (**A**). Different letters showed significant differences (Tukey’s test; *p* ≤ 0.05). Interaction graph of PGPR and CB for soil EC (**B**). Correlation graph of PGPR and CB for soil EC (**C**).

Soil EC was significantly affected by treatments under artificially induced lead (Pb) stress. Interaction of PGPR and CB was non-significant but ordinal for soil EC (Fig. 2B). Results showed that PGPR1 + CB, CB, and PGPR2 + CB were significantly decreased soil EC over soil EC of control (Fig. 2A). A significant decrease in soil EC was also noted in PGPR1 and PGPR2 over control. Application of CB remained significantly better over PGPR for decreasing soil EC as compared to control. However, PGPR1 + CB, CB and PGPR2 + CB were non-significantly with each other for soil EC. It was noted that CB showed a significant (0.00001) negative (− 0.9342) correlation, while PGPR showed a non-significant (0.2987) negative (− 0.2593) correlation with soil EC (Fig. 2C). A significant reduction of 24% in soil EC was observed in over control, where PGPR2 + CB was applied.

**Figure 3:**
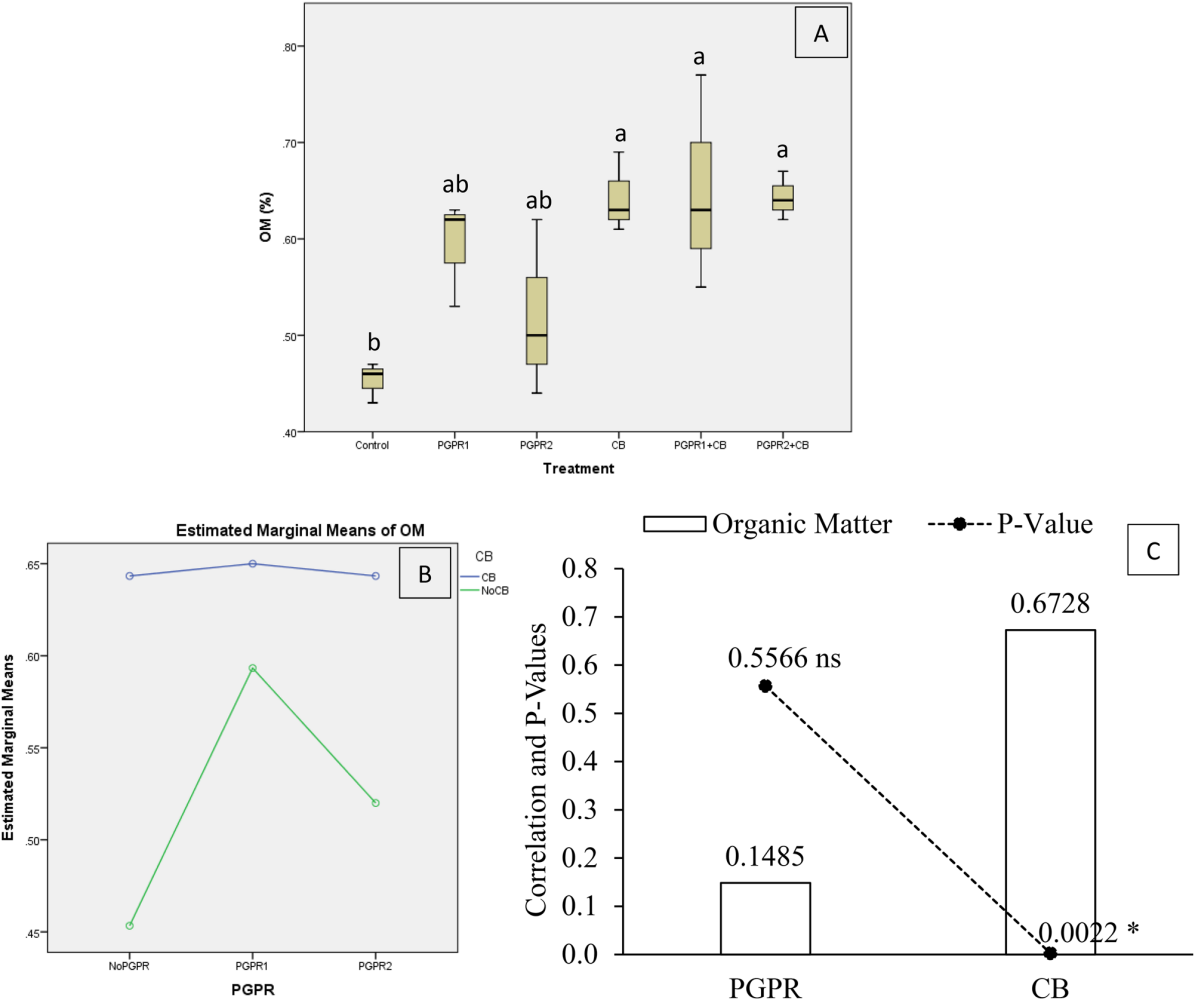
Effect of treatments on the organic matter of the soil. Values are the average of three replicates (**A**). Different letters showed significant differences (Tukey’s test; *p* ≤ 0.05). Interaction graph of PGPR and CB for soil OM (**B**). Correlation graph of PGPR and CB for soil OM (**C**).

Results showed that the treatment's effect was significant on soil organic matter (OM) under artificially induced lead (Pb) stress. Interaction of PGPR and CB was non-significant but ordinal for OM (Fig. 3B). Results showed that CB, PGPR1 + CB and PGPR2 + CB significantly enhanced soil OM over control (Fig. 3A). Sole inoculation of PGPR1 and PGPR2 showed neither a significant increase nor decreased soil OM over control. Application of CB remained significantly better over PGPR1 and PGPR2 for improving the OM over control. However, CB, PGPR1 + CB and PGPR2 + CB did not differ significantly from each other for OM. It was noted that CB showed significant (0.0022) positive (0.6728) correlation while PGPR showed non-significant (0.5566) positive (0.1485) correlation with OM (Fig. 3C). A significant increase of 44% in soil OM was observed in over control where PGPR1 + CB was applied.

**Figure 4:**
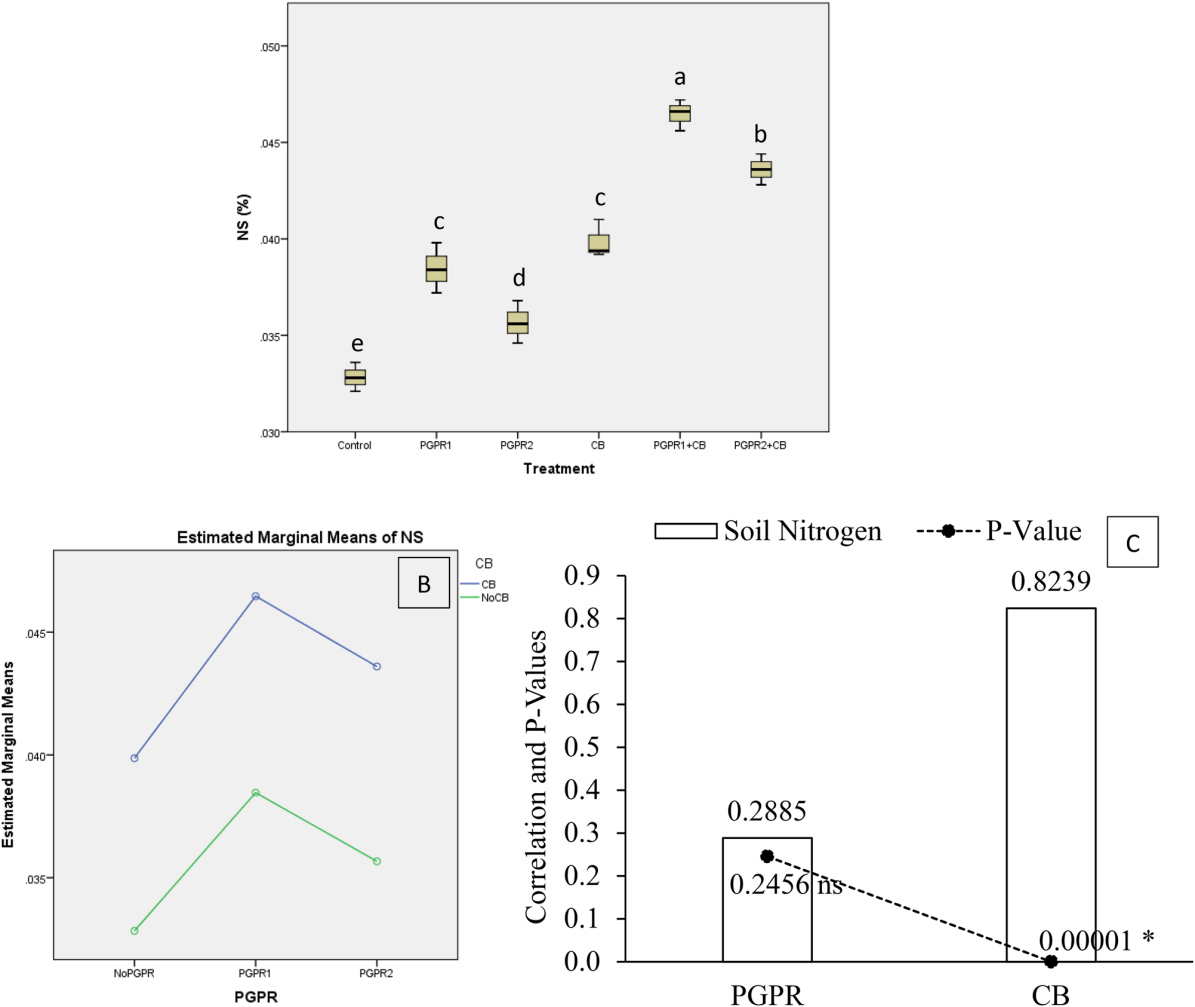
Effect of treatments on soil nitrogen (NS). Values are the average of three replicates (**A**). Different letters showed significant differences (Tukey’s test; *p* ≤ 0.05). Interaction graph of PGPR and CB for soil NS (**B**). Correlation graph of PGPR and CB for soil NS (**C**).

All the treatments significantly affect soil nitrogen (NS) under artificially induced lead (Pb) stress. Interaction of PGPR and CB was non-significant but ordinal for NS (Fig. 4B). Results showed that PGPR1 + CB significantly enhanced NS over control (Fig. 4A). Application of PGPR2 + CB also gave significantly higher NS over CB and control. Treatments PGPR1 and PGPR2 differed significantly over control for NS. Application of CB remained significantly better over PGPR2 but statistically alike with PGPR1 for improving the NS over control. It was noted that CB showed significant (0.00001) positive (0.8239) correlation while PGPR showed non-significant (0.2456) positive (0.2885) correlation with NS (Fig. 4C). A significant increase of 42% in NS was observed in over control, where PGPR1 + CB was applied.

**Figure 5:**
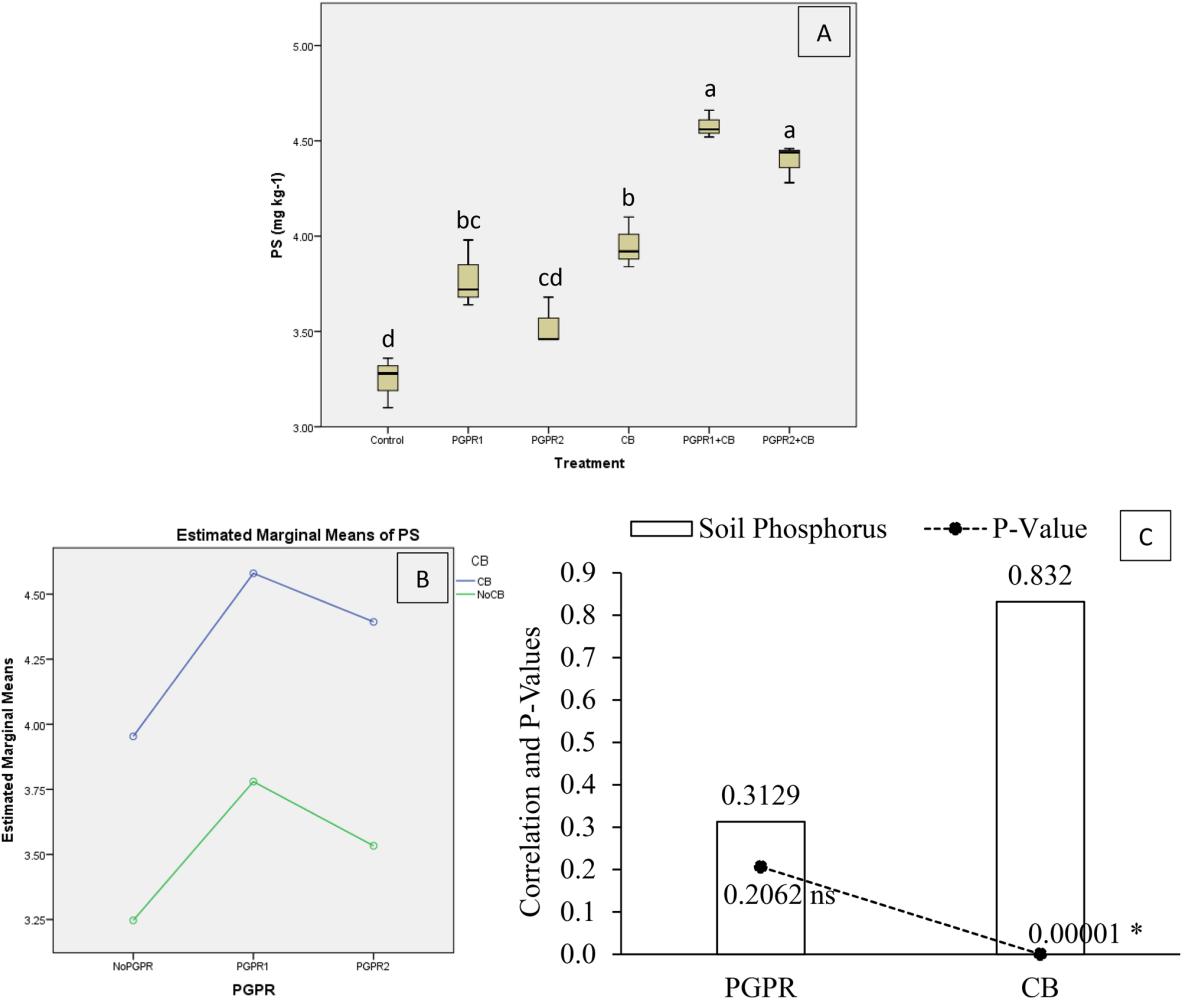
Effect of treatments on soil phosphorus (PS). Values are the average of three replicates (**A**). Different letters showed significant differences (Tukey’s test; *p* ≤ 0.05). Interaction graph of PGPR and CB for soil PS (**B**). Correlation graph of PGPR and CB for soil PS (**C**).

Results indicated that treatments significantly affect soil phosphorus (PS) under artificially induced lead (Pb) stress. Interaction of PGPR and CB was non-significant but disordinal for soil PS (Fig. 5B). Treatments PGPR1 + CB and PGPR2 + CB were significant over control for PS (Fig. 5A). Application of PGPR1 and CB significantly increased PS over control. Treatments PGPR1 gave significantly high PS, but PGPR2 remained non-significant over control. It was noted that CB showed significant (0.00001) positive (0.8320) correlation while PGPR showed non-significant (0.2062) positive (0.3129) correlation with PS (Fig. 5C). A significant increase of 41% in PS was observed in over control, where PGPR1 + CB was applied.

**Figure 6:**
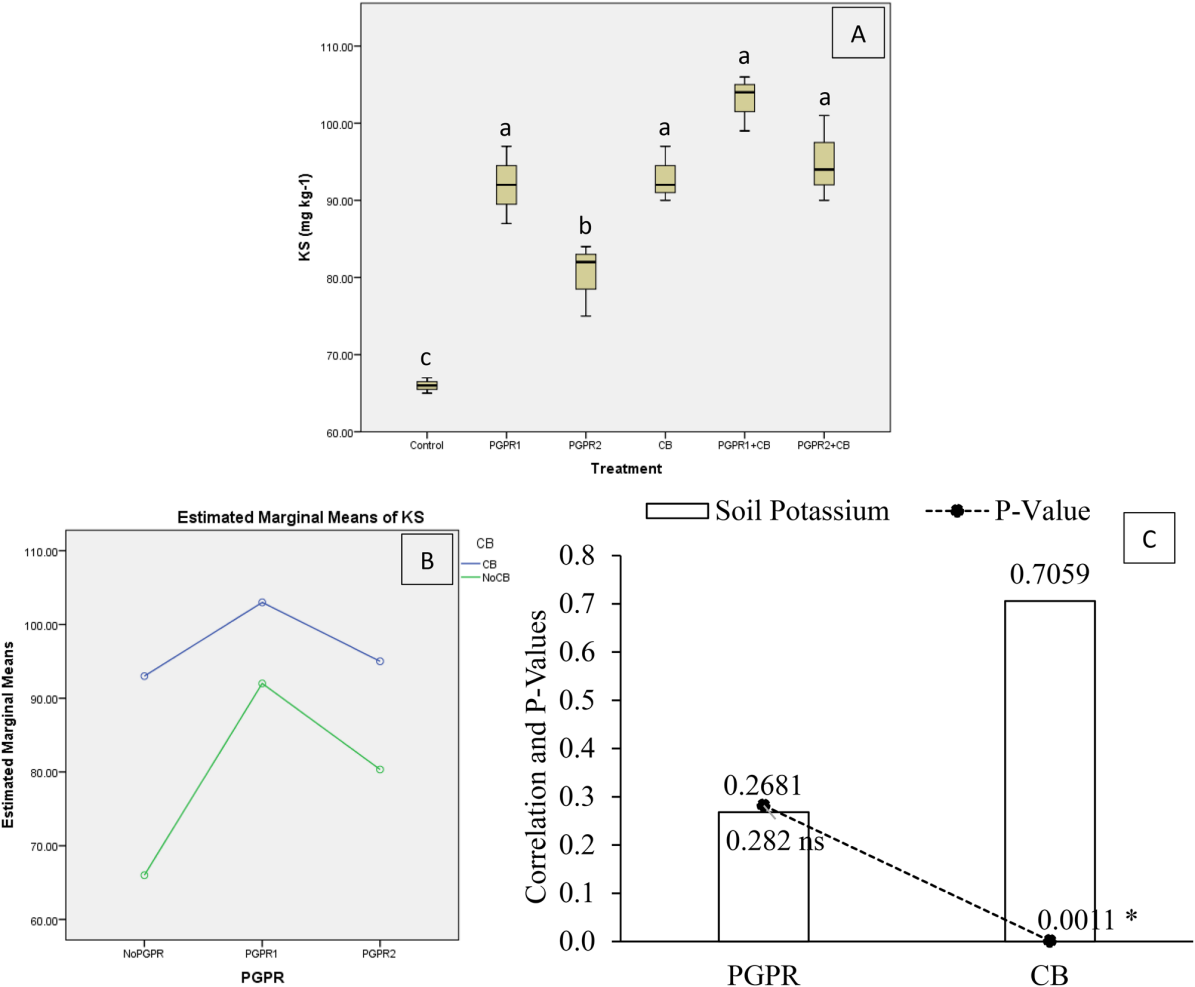
Effect of treatments on soil potassium (KS). Values are the average of three replicates (**A**). Different letters showed significant differences (Tukey ‘s test; *p* ≤ 0.05). Interaction graph of PGPR and CB for soil KS (**B**). Correlation graph of PGPR and CB for soil KS (**C**).

Results indicated that treatments significantly affect soil potassium (KS) under artificially induced lead (Pb) stress. Interaction of CB with PGPR was significant ordinal for KS (Fig. 6B). Application of PGPR1, PGPR1 + CB, CB and PGPR2 + CB were non-significant with each other but gave a significant increase in KS than control (Fig. 6A). Treatment PGPR2 also showed significantly high KS over control. It was noted that CB showed significant (0.0011) positive (0.7059) correlation while PGPR showed non-significant (0.2820) positive (0.2681) correlation with KS (Fig. 6C). A significant increase of 56% in KS was observed in over control, where PGPR1 + CB was applied.

**Figure 7:**
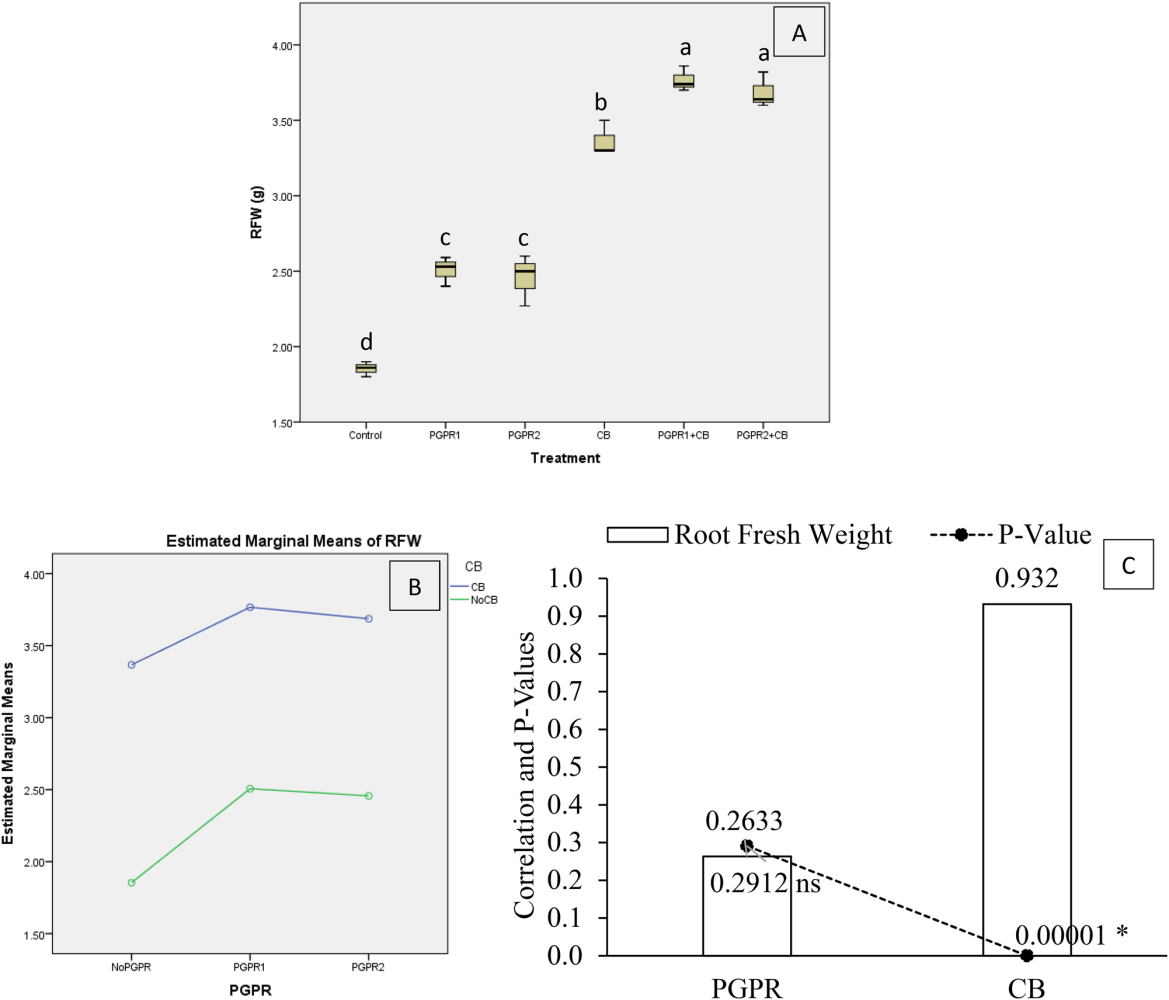
Effect of treatments on mint plants roots fresh weight (RFW). Values are the average of three replicates (**A**). Different letters showed significant differences (Tukey’s test; *p* ≤ 0.05). Interaction graph of PGPR and CB for RFW (**B**). Correlation graph of PGPR and CB for RFW (**C**).

The effect of treatments was significant on mint plants root fresh weight (RFW) under artificially induced lead (Pb) stress. Interaction of PGPR and CB was non-significant but ordinal for soil RFW (Fig. 7B). Treatments PGPR1 + CB and PGPR2 + CB significantly enhanced RFW over control (Fig. 7A). Sole application of CB gave significantly high RFW from control. Inoculation of PGPR1 and PGPR2 also showed a significant increase in RFW over control. It was noted that CB showed significant (0.00001) positive (0.9320) correlation while PGPR showed non-significant (0.2912) positive (0.2633) correlation with RFW (Fig. 7C). A significant increase of 1.03-fold in RFW was observed in over control where PGPR1 + CB was applied.

**Figure 8:**
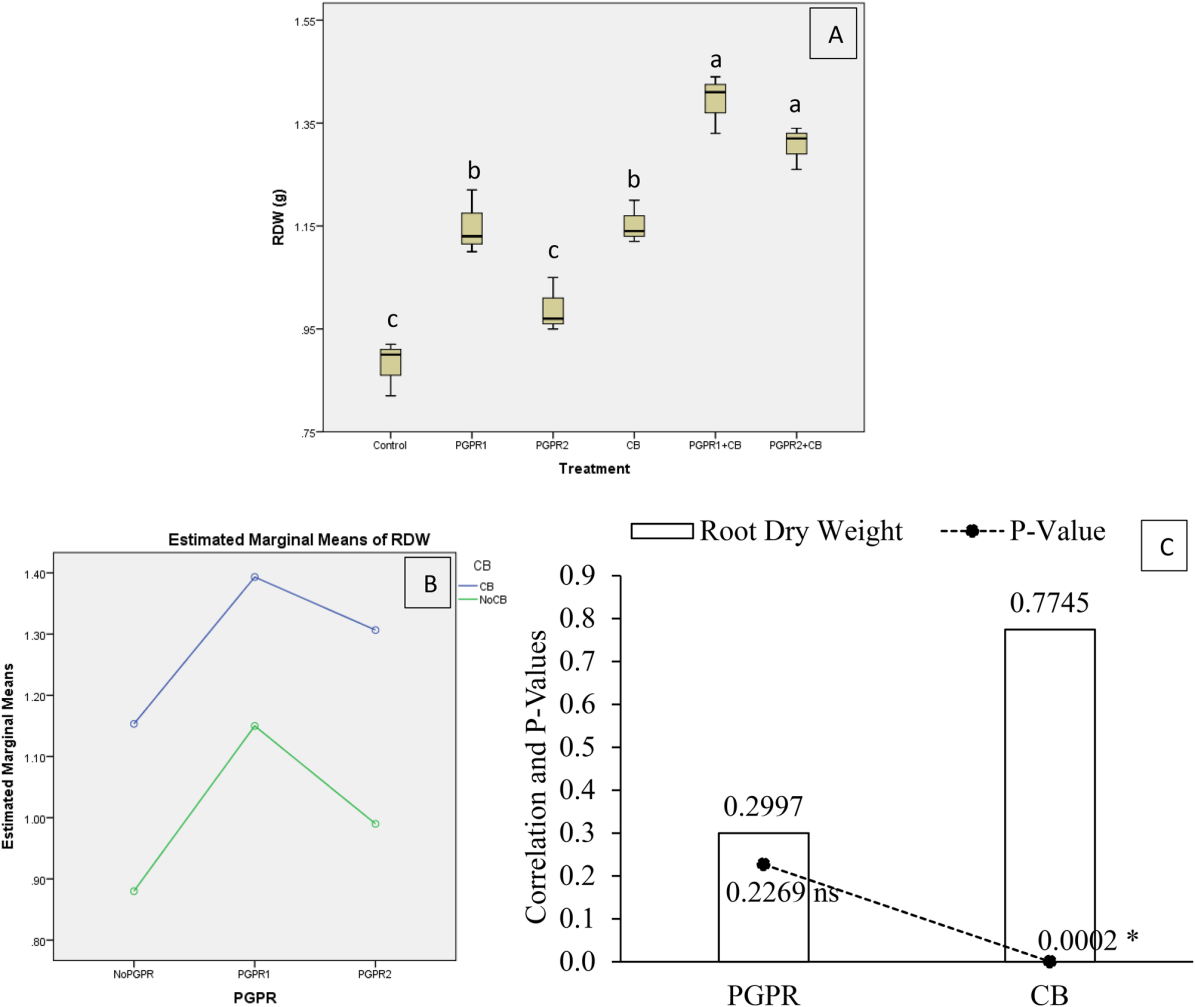
Effect of treatments on mint plants root dry weight (RDW). Values are the average of three replicates (**A**). Different letters showed significant differences (Tukey’s test; *p* ≤ 0.05). Interaction graph of PGPR and CB for RDW (**B**). Correlation graph of PGPR and CB for RDW (**C**).

Application of treatments significantly affects the mint plant's root dry weight (RDW) under artificially induced lead (Pb) stress. Interaction of PGPR and CB was non-significant but ordinal for RDW (Fig. 8B). Treatments PGPR1 + CB and PGPR2 + CB gave a significant increase in RDW over control (Fig. 8A). Treatments PGPR2 was non-significant over control for RDW. Inoculation of PGPR1 and CB application remained statistically alike but gave a significant increase in RDW over control. It was noted that CB showed significant (0.0002) positive (0.7745) correlation while PGPR showed non-significant (0.2269) positive (0.2997) correlation with RDW (Fig. 8C). A significant increase of 58% in RDW was observed in over control, where PGPR1 + CB was applied.

**Figure 9:**
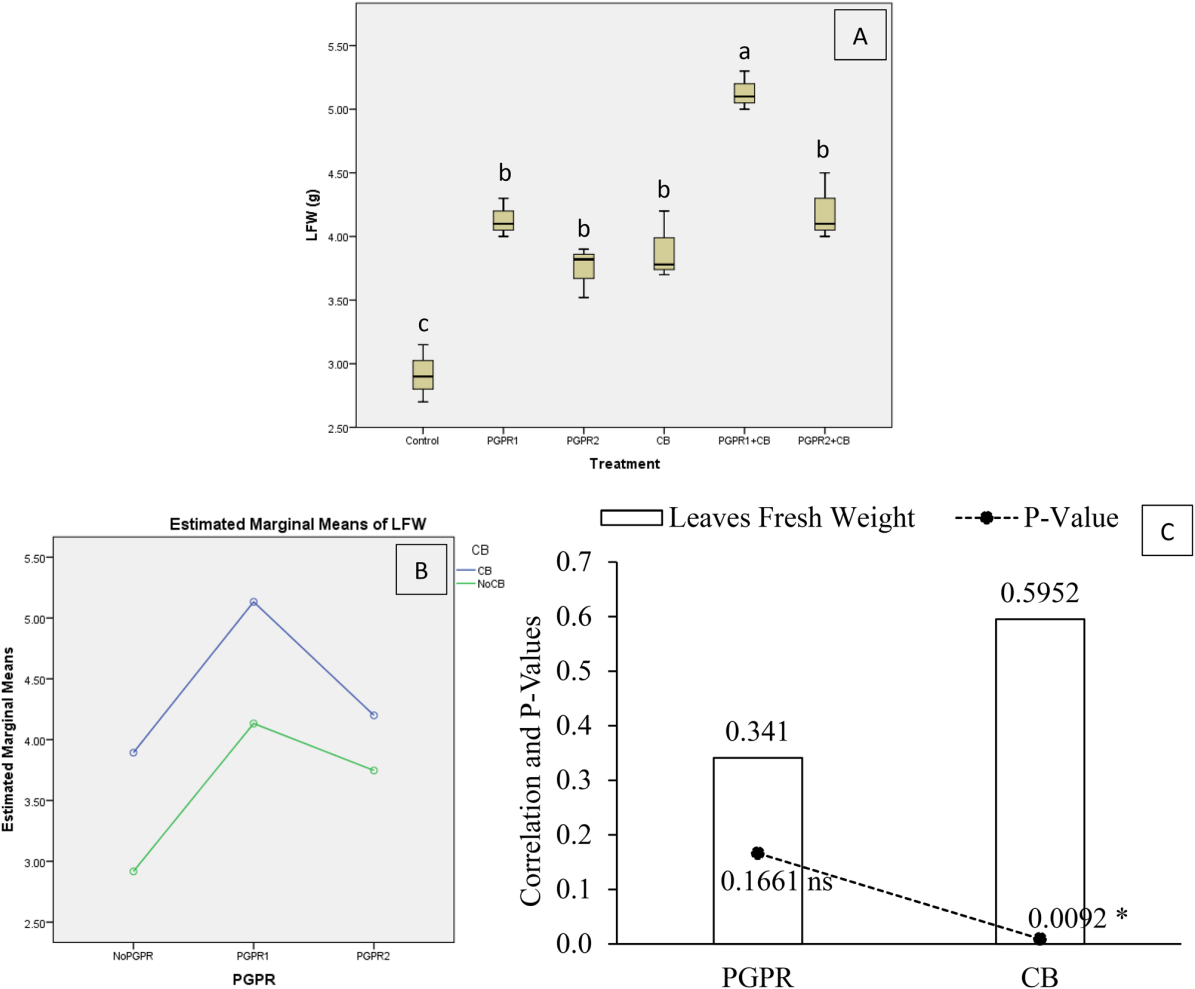
Effect of treatments on mint plants leaves fresh weight (LFW). Values are the average of three replicates (**A**). Different letters showed significant differences (Tukey’s test; *p* ≤ 0.05). Interaction graph of PGPR and CB for LFW (**B**). Correlation graph of PGPR and CB for LFW (**C**).

The addition of treatments significantly affects mint leaves fresh weight (LFW) under artificially induced lead (Pb) stress. Interaction of PGPR and CB was non-significant but ordinal for LFW (Fig. 9B). Treatments PGPR1 + CB significantly increased LFW over control (Fig. 9A). Application of PGPR1, PGPR2, CB and PGPR2 + CB gave significant enhancement in LFW over control. It was noted that CB showed significant (0.0092) positive (0.5952) correlation while PGPR showed non-significant (0.1661) positive (0.3410) correlation with LFW (Fig. 9C). A significant increase of 76% in LFW was observed in over control, where PGPR1 + CB was applied.

**Figure 10:**
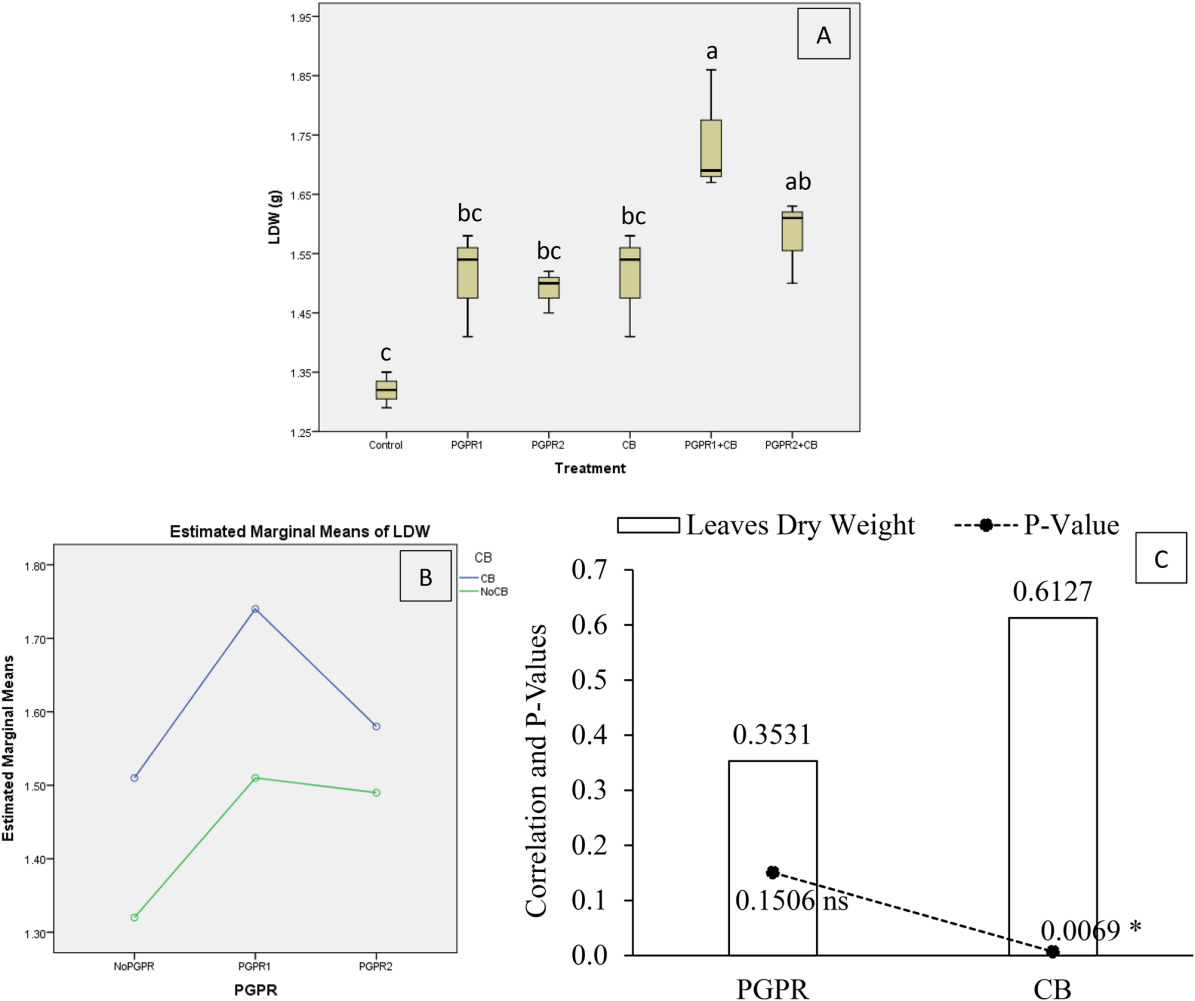
Effect of treatments on mint plants leaves dry weight (LDW). Values are the average of three replicates (**A**). Different letters showed significant differences (Tukey’s test; *p* ≤ 0.05). Interaction graph of PGPR and CB for LDW (**B**). Correlation graph of PGPR and CB for LDW (**C**).

Results showed that treatment's application remained significantly different for mint leaves dry weight (LDW) under artificially induced lead (Pb) stress. Interaction of PGPR and CB was non-significant but ordinal for LDW (Fig. 10B). Treatments PGPR1 + CB and PGPR2 + CB remained statistically alike but increased LDW significantly over control (Fig. 10A). Inoculation of PGPR1, PGPR2 and CB remained non-significant over control for LDW. It was noted that CB showed significant (0.0069) positive (0.6127) correlation while PGPR showed non-significant (0.1506) positive (0.3531) correlation with LDW (Fig. 10C). A significant increase of 32% in LDW was observed in over control where PGPR1 + CB was applied.

**Figure 11:**
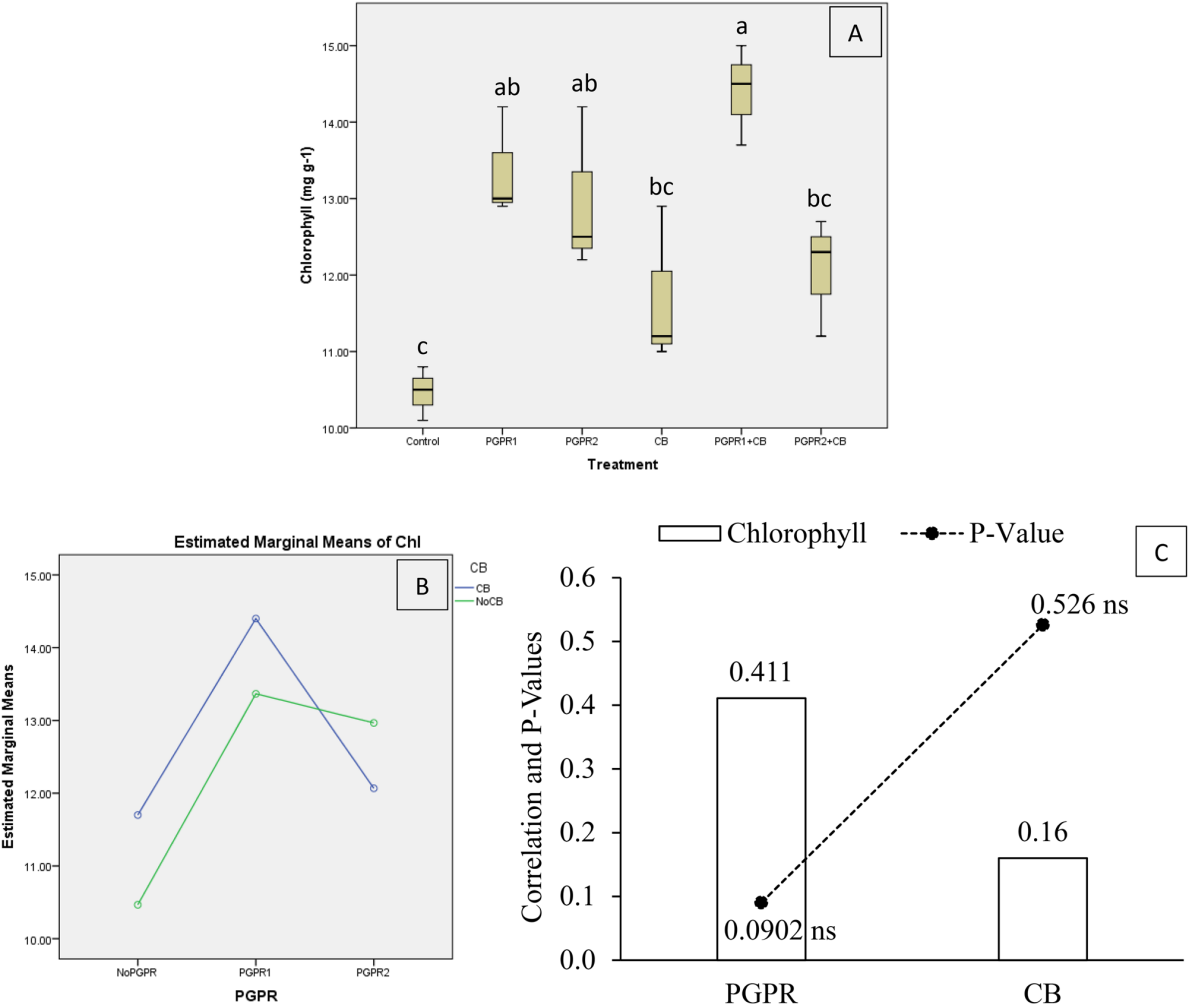
Effect of treatments on mint plants leaves chlorophyll contents. Values are the average of three replicates (**A**). Different letters showed significant differences (Tukey’s test; *p* ≤ 0.05). Interaction graph of PGPR and CB for Chl (**B**). Correlation graph of PGPR and CB for Chl (**C**).

Results showed that treatment's application remained significantly different for mint chlorophyll contents (Chl) under artificially induced lead (Pb) stress. Interaction of PGPR and CB was significant ordinal for Chl (Fig. 11B). Treatments PGPR1 + CB, PGPR1 and PGPR2 increase Chl significantly over control (Fig. 11A). Application of PGPR2 + CB and CB remained non-significant over control for Chl. It was noted that CB showed non-significant (0.5260) positive (0.1600) correlation while PGPR showed non-significant (0.0902) positive (0.4110) correlation with Chl (Fig. 11C). A significant increase of 37% in Chl was observed in over control, where PGPR1 + CB was applied.

**Figure 12:**
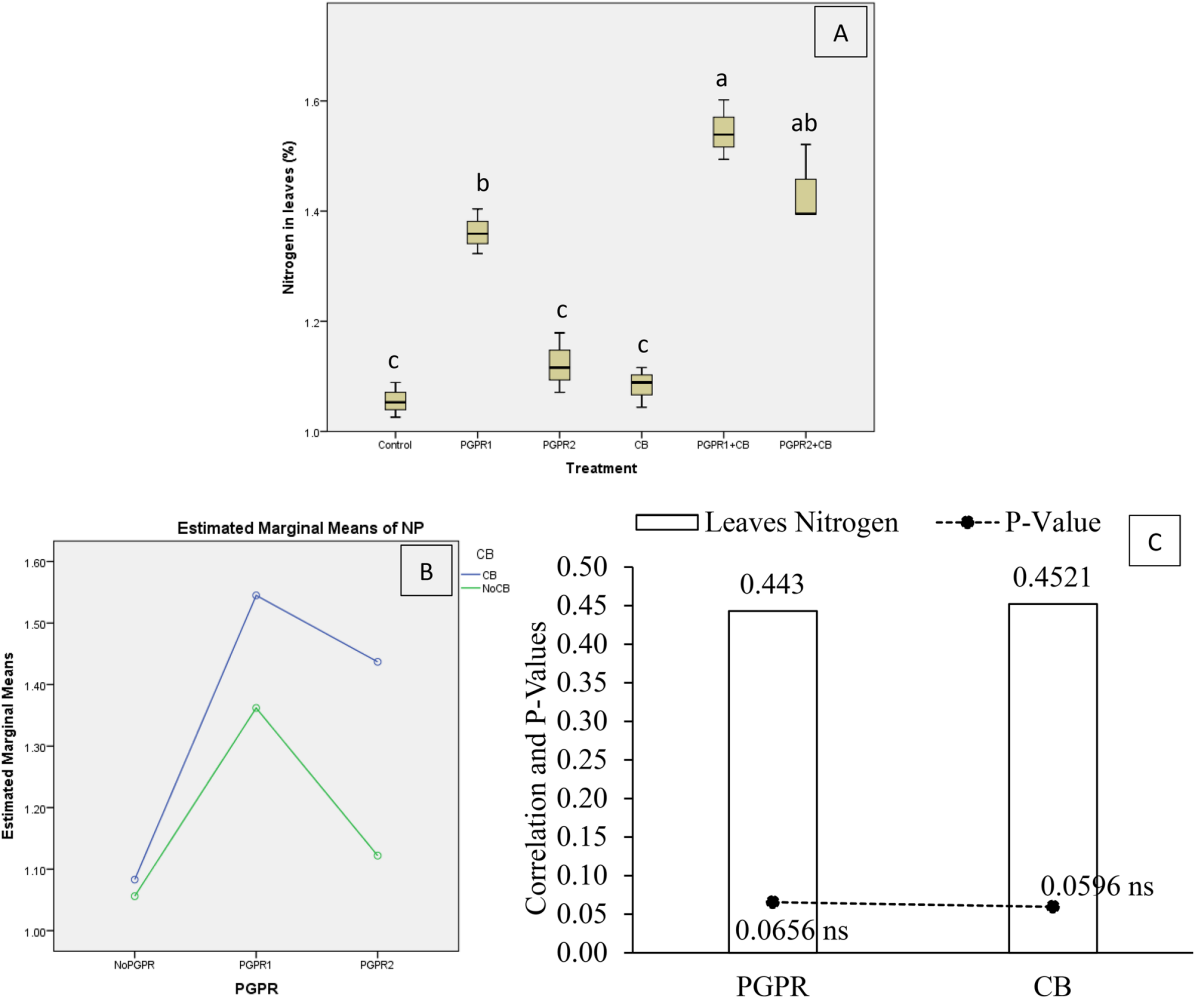
Effect of treatments on mint plants leaves nitrogen concentration (NP). Values are the average of three replicates (**A**). Different letters showed significant differences (Tukey’s test; *p* ≤ 0.05). Interaction graph of PGPR and CB for NP (**B**). Correlation graph of PGPR and CB for NP (**C**).

Nitrogen concentration in mint leaves (NP) was significantly affected by treatments under artificially induced lead (Pb) stress. Interaction of PGPR and CB was significant ordinal for NP (Fig. 12B). Treatments PGPR1 + CB and PGPR2 + CB remained statistically alike but significantly increase NP over control (Fig. 12A). Application of CB and PGPR2 remained non-significant over control for NP. However, sole inoculation of PGPR1 gave a significant increase in NP over control. It was noted that CB showed non-significant (0.0596) positive (0.4521) correlation while PGPR showed non-significant (0.0656) positive (0.4430) correlation with NP (Fig. 12C). A significant increase of 46% in NP was observed in over control, where PGPR1 + CB was applied.

**Figure 13:**
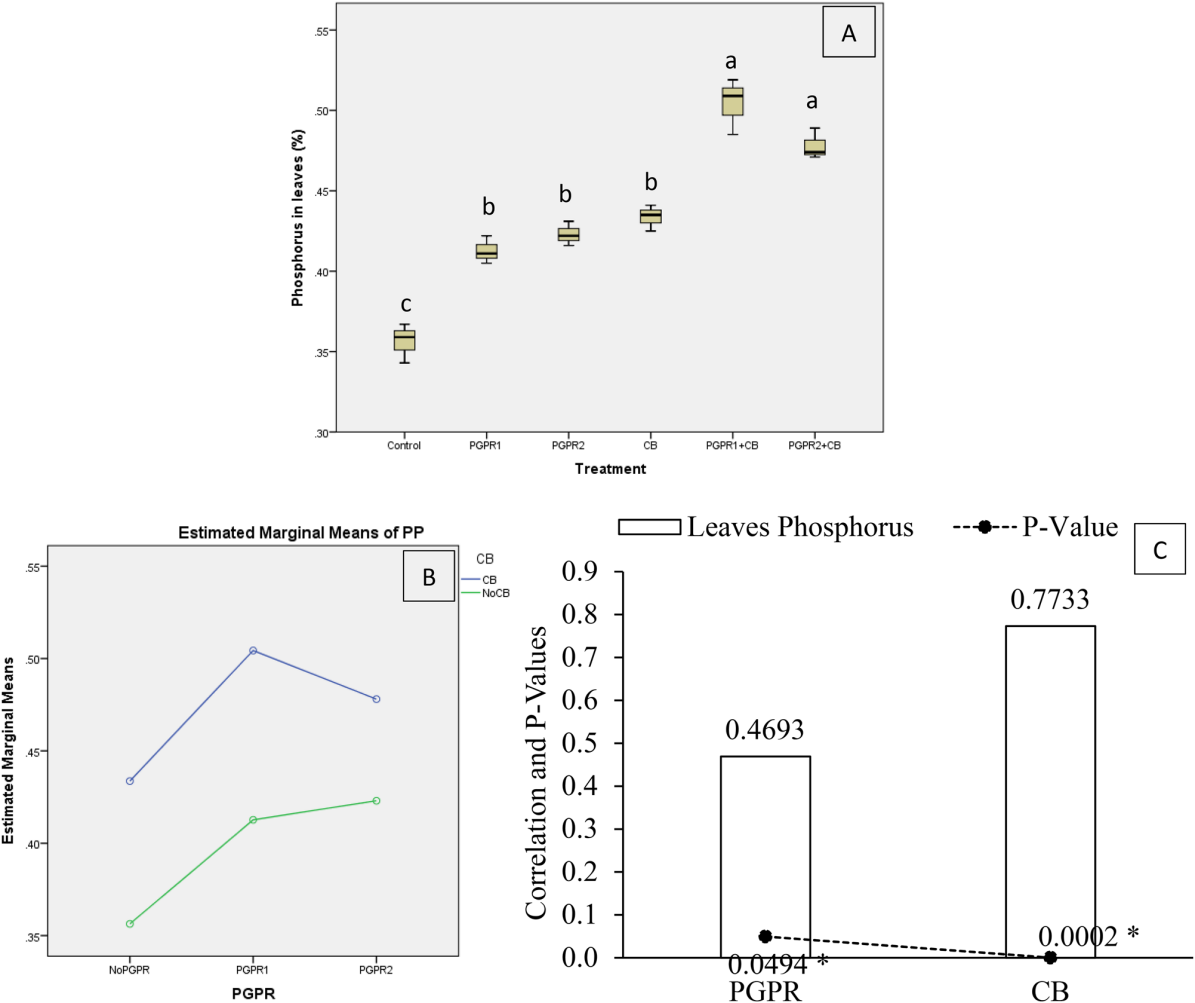
Effect of treatments on mint plants leaves phosphorus concentration (PP). Values are the average of three replicates (**A**). Different letters showed significant differences (Tukey’s test; *p* ≤ 0.05). Interaction graph of PGPR and CB for soil PP (**B**). Correlation graph of PGPR and CB for soil PP (**C**).

Phosphorus concentration in mint leaves (PP) was significantly affected by treatments under artificially induced lead (Pb) stress. Interaction of PGPR and CB was significant ordinal for PP (Fig. 13B). The application of PGPR1 + CB and PGPR2 + CB remained non-significant but increased PP significantly over control (Fig. 13A). However, treatments PGPR1, PGPR2 and application of CB also remained significant for PP over control. It was noted that CB showed significant (0.0002) positive (0.7733) correlation while PGPR also showed significant (0.0494) positive (0.4693) correlation with PP (Fig. 13C). A significant increase of 39% in PP was observed in over control, where PGPR1 + CB was applied.

**Figure 14:**
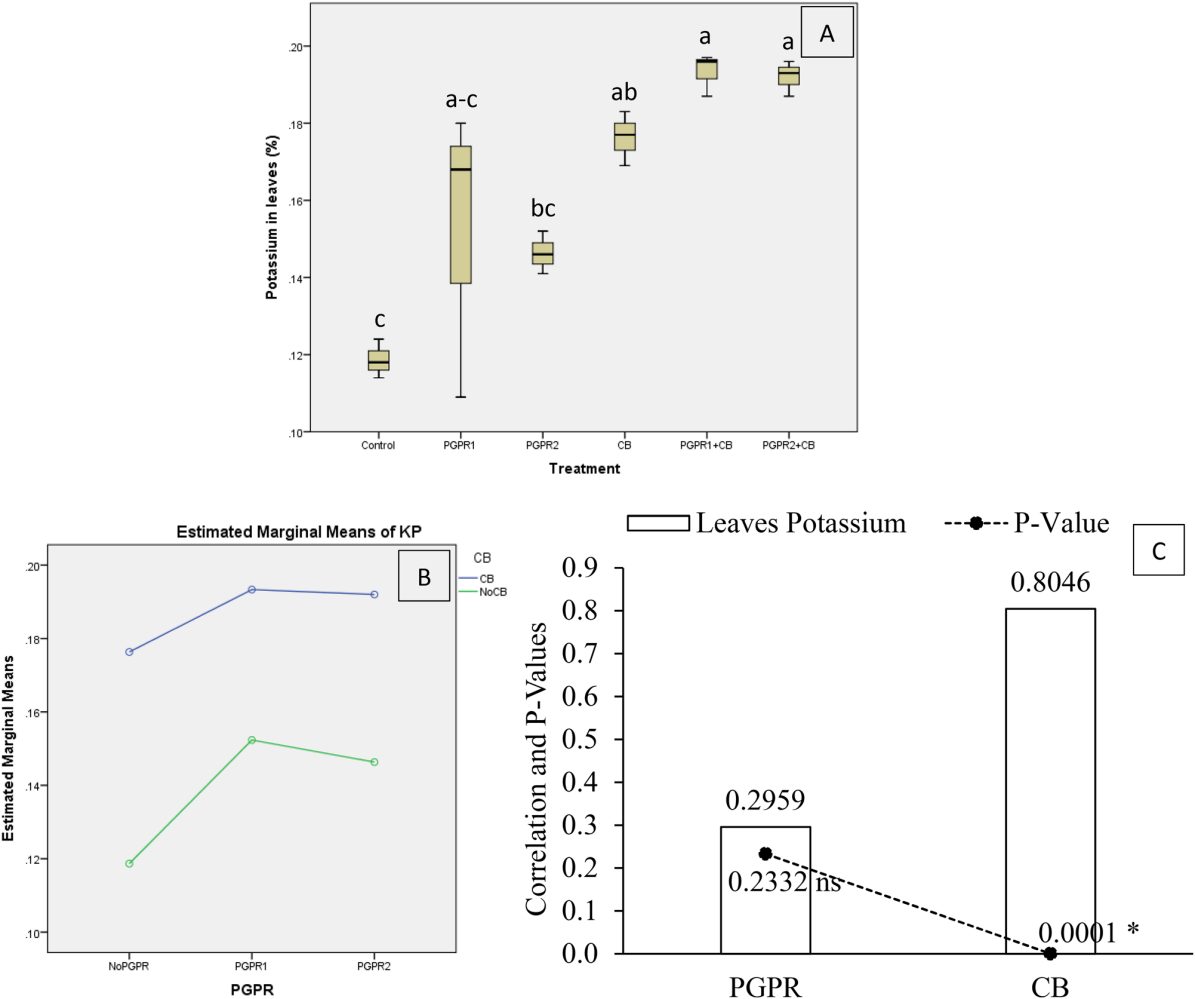
Effect of treatments on mint plants leaves potassium concentration (KP). Values are the average of three replicates (**A**). Different letters showed significant differences (Tukey’s test; *p* ≤ 0.05). Interaction graph of PGPR and CB for KP (**B**). Correlation graph of PGPR and CB for KP (**C**).

Potassium concentration in mint leaves (KP) was significantly affected by treatments under artificially induced lead (Pb) stress. Interaction of PGPR and CB was non-significant but ordinal for KP (Fig. 14B). Treatments PGPR1 + CB, CB and PGPR2 + CB, increased KP significantly over control (Fig. 14A). However, treatments PGPR2 and PGPR1 also remained non-significant for KP over control. It was noted that CB showed significant (0.0001) positive (0.8046) correlation while PGPR also showed non-significant (0.2332) positive (0.2959) correlation with KP (Fig. 14C). A significant increase of 63% in KP was observed in over control, where PGPR1 + CB was applied.

**Figure 15:**
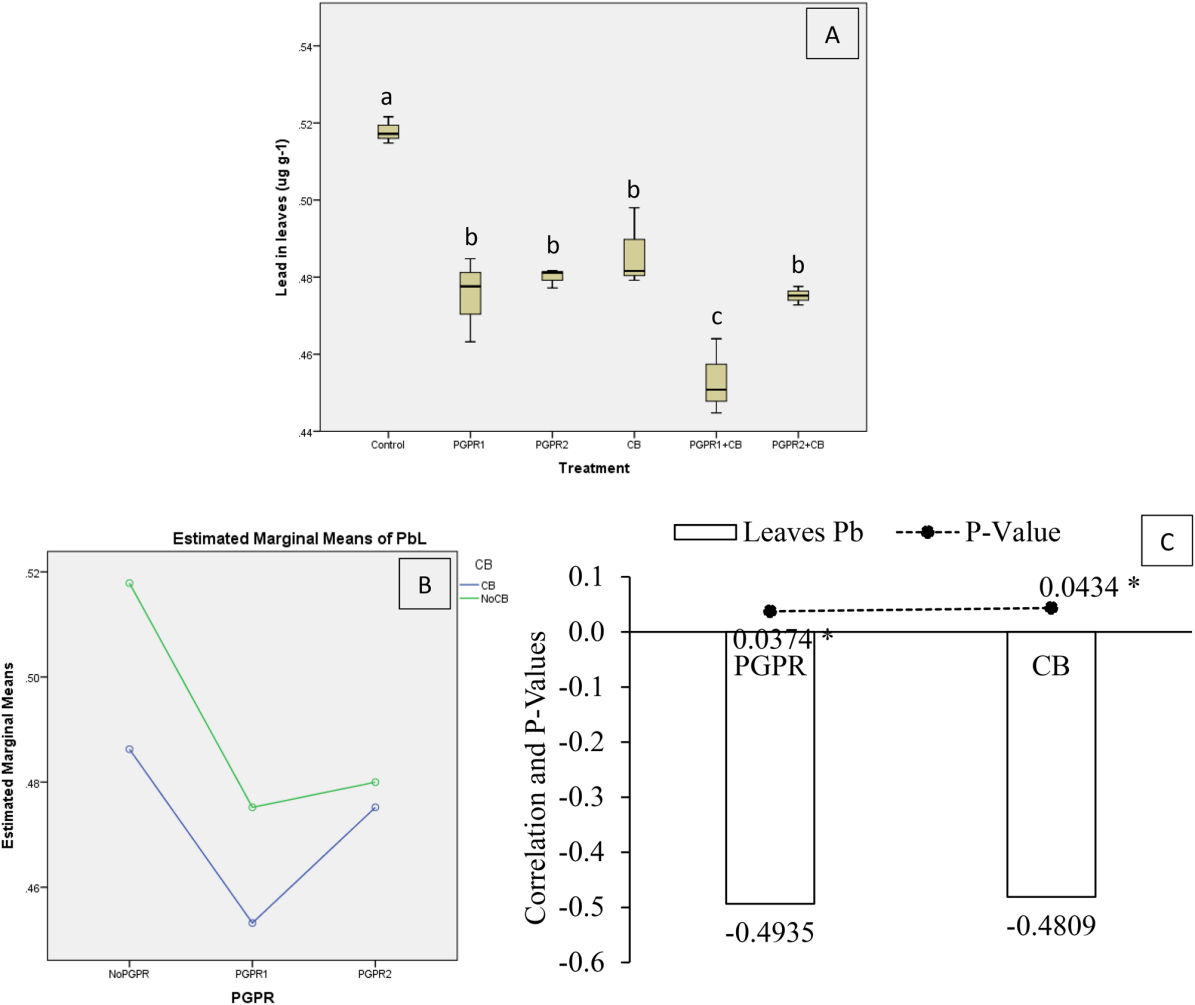
Effect of treatments on mint plants leaves lead concentration (PbL). Values are the average of three replicates (**A**). Different letters showed significant differences (Tukey’s test; *p* ≤ 0.05). Interaction graph of PGPR and CB for PbL (**B**). Correlation graph of PGPR and CB for PbL (**C**).

Lead concentration in mint leaves (PbL) was significantly affected by treatments under artificially induced lead (Pb) stress. Interaction of PGPR and CB was non-significant but ordinal for PbL. Application of PGPR1 + CB significantly decreased PbL over control (Fig. 15A,B). However, sole inoculation of PGPR2, PGPR1, CB and PGPR2 + CB decreased PbL significantly over control. It was noted that CB showed a significant (0.0434) negative (− 0.4809) correlation, while PGPR also showed a significant (0.0374) negative (− 0.4935) correlation with PbL (Fig. 15C). A significant decrease of 13.5% in PbL was observed in over control, where PGPR1 + CB was applied.

## Discussion

In the current study, the application of CB with and without PGPR decreased soil pH. This decrease in soil pH was due to the low pH of compost and biochar over the soil. Also, the secretion of organic acids by rhizobacteria played an imperative role in decreasing soil pH. Most of the chemical properties of biochar are dependent on the nature of feedstock^26,62^. Combined application of organic amendments and biochar catalyzed the oxidation processes^63^. A significant enhancement in biochar oxidation played an efficacious role in releasing the carboxylic functional group in soil. This carboxylic functional group decrease soil pH thus, regulate the exchange of mineralized ions^64,65^. Many rhizobacteria in soil secrete organic acids, which also act as an allied factor for decreasing soil pH^66^. Besides biochar, the decomposition of compost in the soil also releases acidic compounds^67^. The presence of a high concentration of humic acid in composted materials also efficaciously played its role in decreasing soil pH^68^.

Furthermore, compost has readily degradable carbon compounds by microbes of the rhizosphere by acidic secretions. These compounds are soluble in water and contribute to the decrease of soil pH^69^. Biochar has the potential to enhance the soil ions exchangeability^70^.

Enhance cation exchange capacity, in turn, increases ion retention in the rhizosphere. As a result, EC*e* of the soil is increased^71^. Furthermore, colonization of PGPRs enhances the root's surface area, which facilitates the plants regarding better nutrients uptake^72^. Under partial or no supply of oxygen and high-temperature combustion caused carbon sequestration, which yields activated carbon (biochar)^62^. Better soil aggregation after biochar addition facilitates soil OM buildup^71–75^. It also enhances soil microbial proliferation and activities in the rhizosphere^76,77^. Also, the use of compost in the current study was an allied factor for a significant increase in soil organic matter. Continuous addition of compost as amendments significantly improves soil organic matter on a long-term basis^78^. Besides, biochar can also control soil nutrients losses by leaching^79^. Better retention of soil nutrients due to the high exchangeability of biochar improves soil's fertility status^80,81^. PGPR secrets different organic acids (tartaric acid, oxalic acid, malic acid, citric acid, succinic acid) that modify soil pH. Siderophores produced by PGPR actively chelate potassium ions and enhances their bioavailability to the crops^82,83^.

A major part of compost also contributes to the provision of mineralized K that is an allied factor for improving soil health and fertility level^84^. When applied in combination with biochar, organic manure modify the plant's roots physiology, facilitating better nutrient availability^85^. Growth hormones, i.e., indole acetic acid (IAA) secretion by PGPR, also enhance roots elongation^86^. The results of the current study also support the above findings. Both rhizobacteria used in the current study were capable of producing IAA growth hormone that played an imperative role in significant plant growth improvement. Mohite^87^ suggested that IAA increases the growth of adventitious roots. These roots are directly involved in nutrients uptake. Compost and biochar addition in the soil thus ameliorate soil properties and increase soil fertility level linked with significant improvement in biomass production of crops^88,89^. Danish and Zafar-ul-Hye^42^ also noted the efficiency of rhizobacteria is increased when inoculated with biochar^43^. A significant improvement in N, P and K concentration of mint leaves also validated such results. Pore spaces and exchange sites of compost mixed biochar in the current study effectively enhanced the bioavailability of nutrients to the mint plants. In addition to the above, a significant decrease in soil pH also played a vital in the mobilization of fixed P. High contents of K in compost has also contributed to improved soil pH regarding enhancement in the K uptake in mint plants. Accumulation of stress generating ethylene in plants under toxicity of heavy metals also deteriorates crops' growth and yield. This ethylene is decomposed into α-ketobutyrate and ammonia by ACC deaminase produced by PGPRs, resulting in alleviation of stress induced by heavy metals^90,91^. Zafar-ul-Hye et al.^41^ also documented similar findings by using ACC deaminase producing PGPR under heavy metal toxicity. The compost application also helped in the provision of energy to PGPRs and enhance oxygen transfer, which facilitates the immobilization of metallic ions in soil^92^. As both rhizobacteria of the current study were also capable of producing ACC deaminase, they also act as an allied factor for the improvement in mint growth under Pb toxicity. Song and Greenway^93^ also observed that heavy metals become bounded with the compost's exchange sites in the soil. The presence of surface-active function in biochar sorp the heavy metals electrostatically thus caused their immobilization in soil^94^. Among different functional groups for heavy metals, immobilization through biochar CO_3_^−2^ and hydroxides are predominant^95,96^. Change in redox potential and rhizosphere acidification via PGPRs secretions, the bioavailability of heavy metals to plants is also decreased^53,55,97,98^.

## Conclusion

It is concluded that both CB and *A. faecalis* treatments effectively minimize the Pb toxicity in min Plants. However, the use of *A. faecalis* + CB as a treatment is a better approach than the sole application of CB and *A. faecalis* under Pb toxicity for improvement in growth attributes, nutrients concentration and mitigation of Pb toxicity in mint. More investigations are suggested to introduce *A. faecalis* + CB as an efficient treatment for alleviating Pb stress in the mint at field levels.

## Materials and methodology

### Treatments preparation

From Sabzi Mandi, Multan, fruit and vegetable waste were collected for the manufacturing of biochar. To achieve < 15% moisture sun-drying of waste material was done for 14 days. After sun drying, small pieces of waste material were put in pyrolyzer at the temperature of 450 °C and pyrolyzed for 2 h under the partial oxygen presence. After that pyrolyzer drum was left for cooling. Finally, biochar was grinded and pass through 2 mm sieve. To make organic amendment (compost mixed biochar), compost was purchase from Buraq Agro Chemicals, Industrial State Area, Multan. For experimental purposes, biochar was mixed with compost in 1:1 ratio and applied in the soil at the rate of 0.5% (5 g kg^−1^). Application of compost mixed biochar was done at the time of pot filling with soil as per treatment plan. PGPRs i.e., *Alcaligenes faecalis* and *Bacillus amyloliquefaciens* were collected from Soil Microbiology and Biochemistry Laboratory, BZU, Multan and propagated in Dworkin and Foster (DF) media^99^. The inoculation of mint seeds was done using inoculum 0.5 nm optical density of inoculum (5 ml 100 g^−1^ seeds). The final top dressing was done with sterilized peat, clay and sugar solution (10%). Inoculation of PGPR was done before 30 min of sowing.

### Experimental organization

A pot study was carried on the experimental farm of the Faculty of Agricultural Sciences and Technology. Table 1 has a pre-experimental soil characterization.

**Table 1:** Analyses of compost, soil and rhizobacteria (Pre-sowing)^55^.

Characteristics	Soil	Compost	Biochar	Characteristics	*A. faecalis*	*B. amyloliquefaciens*
Textural class	Loam	–	–	IAA L-Tryptophan (µgml^−1^)	15.33	22.23
pH_***s***_	8.30	5.3	8.04			
EC_***e***_ (dS m^−1^)	1.25	–	3.49			
OM (%)	0.30	–	–	IAA (No L-Tryptophan (µg ml^−1^)	2.21	5.63
Total N (%)	0.015	1.00	1.63			
Available phosphorus (µg g^−1^)	4.62	0.53	0.40			
Extractable potassium (µg g^−1^)	70	55	27	ACC deaminase α-ketobutyrate nmol g^−1^protein h^−1^ Exopolysaccharide	484	232
Extractable lead (µg g^−1^)	0.50	1.15	2.09			
Volatile matter (%)	–	–	14.4			
Ash content (%)	–	–	16.8		+	+
Fixed carbon (%)	–	–	68.8	Phosphate solubilization	+	+

## Treatments

Total six treatments were applied in 3 replications following a complete randomized design (CRD). The treatments were controlled, PGPR1 (*A. faecalis*), PGPR2 (*B. amyloliquefaciens*), 1:1 compost mixed biochar (CB), PGPR1 + CB and PGPR2 + CB. Each pot was filled with 7 kg of soil, and 15 seeds of mint were sown. After germination, only five seedlings were maintained by thinning. Macronutrients were applied at the rate of 33 (K), 80 (P), and 130 (N) kg ha^−1^, in the form of sulphate of potash, Nitrophos and Calcium Ammonium Nitrate at the time of pot preparation. After one week of germination and thinning, Pb stress was applied artificially. Lead sulphate (PbSO_4_) was applied at 250 mg kg^−1^ soil for inducing lead stress^100^.

### Data collection

#### Soil analyses

Bouyoucos hydrometer was used for the determination of soil textural class^101^. The pH of saturated paste was determined pre-calibrated pH meter. Electrical conductivity (EC) was assessed on a pre-calibrated EC meter. Walkley and Black^102^ method was used for soil organic matter determination. Olsen extraction method was adopted for the determination of soil extractable P^103^. Ammonium acetate was used to extract soil potassium, and K was assessed using a flame photometer^104^.

#### Chlorophyll contents

Fresh leaves were taken and cut into small pieces, and 0.5 g leaf samples were immersed in 10 ml acetone for 24 h. Extract of chlorophyll was measured, and color intensity was determined at 645 nm and 663 nm by spectrophotometer^105^. From intensity values, chlorophyll contents were determined by the following formula:

$$\begin{aligned} {\text{Chlorophyll a }}\left( {{\text{mg}}\;{\text{g}}^{ - 1} } \right) & = \left[ {12.7{ } \times {\text{OD}}663 - 2.69 \times {\text{OD}}645} \right] \times \frac{{\text{V}}}{1000} \times {\text{W}} \\ {\text{Chlorophyll b }}\left( {{\text{mg}}\;{\text{g}}^{ - 1} } \right) & = \left[ {22.9{ } \times {\text{OD}}645 - 4.68 \times {\text{OD}}663} \right] \times \frac{{\text{V}}}{1000} \times {\text{W}} \\ {\text{Total chlorophyll }}\left( {{\text{mg}}\;{\text{g}}^{ - 1} } \right) & = {\text{Chl a}} + {\text{Chl b}} \\ \end{aligned}$$

where OD = Optical density (wavelength), V = Final volume made, W = Fresh leaf made (g).

#### Plant analyses

Nitrogen was analyzed on Kjeldhals distillation apparatus^106^. For phosphorus determination, the plant samples were digested in an acid mixture of HNO_3_ and HCIO_4_^107^. The phosphorus was determined by the yellow color method at 470 nm wavelength by using spectrophotometer^106^. For the determination of potassium, the digested sample aliquot was fed to the flamephotometer^104^. The reading of di-acid digested filtrate was noted on atomic absorption spectrophotometer for determination of Pb in leaves^27^.

### Statistical analyses

Analysis of variance was done using SPSS 20, Duncan's (*p* ≤ 0.05) test was applied to compare means among the different groups. Data were analyzed using the standard statistical procedure as followed by Steel et al.^108^.
